# A Novel Naphthyridine Derivative, 3u, Induces Necroptosis at Low Concentrations and Apoptosis at High Concentrations in Human Melanoma A375 Cells

**DOI:** 10.3390/ijms19102975

**Published:** 2018-09-29

**Authors:** Qinghong Kong, Jianxin Lv, Shengjiao Yan, Kwen-Jen Chang, Guanlin Wang

**Affiliations:** 1 Faculty of Life Science and Technology, Kunming University of Science and Technology, Kunming 650500, China; lynk8484@yahoo.co.jp (Q.K.); al-zn@foxmail.com (J.L.); kwenjenchang@yahoo.com (K.-J.C.); 2 School of Chemical Science and Technology, Yunnan University, Kunming 650091, China; 3 Faculty of Enviromental Science and Engineering, Kunming University of Science and Technology, Kunming 650500, China

**Keywords:** naphthyridine derivative, death receptors, apoptosis, caspase-8, necroptosis, melanoma

## Abstract

Naphthyridine derivatives are a widely-used class of heterocycles due to their pharmacological activities. A novel compound (10-Methoxy-1,2,3,4-tetrahydrobenzo(*g*)(1,3) diazepino(1,2-a)-(1,8)naphthyridin-6-yl)(phenyl) methanone (named 3u), showed good anticancer activity in the human malignant melanoma cell line A375 via Thiazolyl Blue Tetrazolium Bromide (MTT) assay. After Western blotting confirmed, we found that 3u induces necroptosis at low concentrations and apoptosis at high concentrations via the upregulation of death receptors and scaffold protein in A375 cells. Furthermore, by combining 3u with the caspase inhibitor zVAD-fmk or Receptor Interacting Serine/Threonine Kinase 1 (RIP1) kinase inhibitor Necrostatin-1 (Nec-1), we found that the activity of caspase-8 was the crucial factor that determined whether either apoptosis or necroptosis occurred. The results indicate that 3u should be considered as a potential chemical substance for melanoma treatment.

## 1. Introduction

Melanoma is a disease besetting human beings for several centuries, and it has been documented in the literature since the 1600s [1]. During the last three decades, morbidity of melanoma has increased [2], and the death rate continues to increase faster than the rate of many cancers [3], whereas that for many other types of tumors have decreased [4]. Epidemiological data show 132,000 new cases of melanoma and 50,000 melanoma-related deaths occurring annually all over the world [5]. For a long time, surgical resection of early tumors represented the sole therapeutic choice, and chemotherapy was introduced only later in the treatment of melanoma [6]. It is already known that one of the hallmarks of cancer is resisting cell death, which is conducive to carcinogenesis and resistance to conventional anticancer therapies [7]. Unfortunately, metastatic melanoma is often resistant to anticancer drugs in common use [8]. Hence, looking for novel anticarcinogens that induce cancer programmed cell death (PCD) pathways is an efficient strategy to suppress cancer.

PCD containing apoptosis, autophagy, and necrosis is defined as regulated cell death mediated by an intracellular program. Apoptosis was originally regarded as the only form of PCD that plays an important role in chemotherapy-induced cancer cell death in many types of cancers [9,10,11]. Two essential pathways exist to induce apoptosis: the extrinsic death receptor pathway and the intrinsic mitochondrial pathway [12]. The extrinsic pathway is initiated by the binding or activating of death receptors (DRs) of the tumor necrosis factor (TNF) receptor superfamily such as TNFR1, Fas, DR4, and DR5 with their extracellular ligands or drugs. This process is followed by the assembly of the death-inducing signal complex (DISC), which contains the Fas-associated via death domain protein (FADD), procaspase-8/10, and/or TNFRSF1A-associated via death domain protein (TRADD). After procaspase-8/10 self-cleavage, DISC then either activates the downstream effector caspases (3, 6, and 7) to directly trigger cell death or cleaves the Bcl-2 family member BH3 Interacting Domain Death Agonist (Bid) into truncated-Bid (t-Bid) to induce the mitochondria-mediated intrinsic apoptotic pathway [13,14]. On the other side, the intrinsic pathway is associated with mitochondrial outer membrane permeabilization (MOMP) under the control of a series of Bcl-2 family members, such as Bcl-2, Bid, and BCL2 Associated X (Bax). Then, cytochrome c is released from mitochondria into the cytosol; it then recruits Apoptotic Peptidase Activating Factor 1 (Apaf-1) and procaspase-9 to compose the apoptosome. The apoptosome results in the cleavage of caspase-9 and then caspase-3, 6, and 7, inducing cell death [15,16].

Necrosis is stereotyped as a form of cell death occurring in an unregulated manner as a result of adverse conditions. Nevertheless, conventional death receptors can trigger necrosis upon inhibition of apoptosis by a caspase inhibitor, zVAD-fmk [17]. This novel form of necrosis, which was given the name ‘necroptosis’ [18], is initiated by activation of the death receptors of the TNFR superfamily. Then, the receptor interaction protein (RIP) family, RIP1 and RIP3, FADD, caspase-8, and/or TRADD assemble together with death receptors. The decision between cell death via apoptosis or necroptosis is determined at this step. Once caspase-8 is activated, it leads to apoptosis via cleaving RIP1 and blocking the functions of RIP1 [19,20]. However, the inhibition of caspase-8 by specific conditions (such as zVAD-fmk) will abrogate the apoptotic signal to favor necroptosis [21,22]. After self-phosphorylation of RIP1, it phosphorylates RIP3. Then, Mixed Lineage Kinase Domain Like Pseudokinase (MLKL) is phosphorylated by the RIP1-RIP3 complex, which induces necroptosis [23].

**Figure 1:**
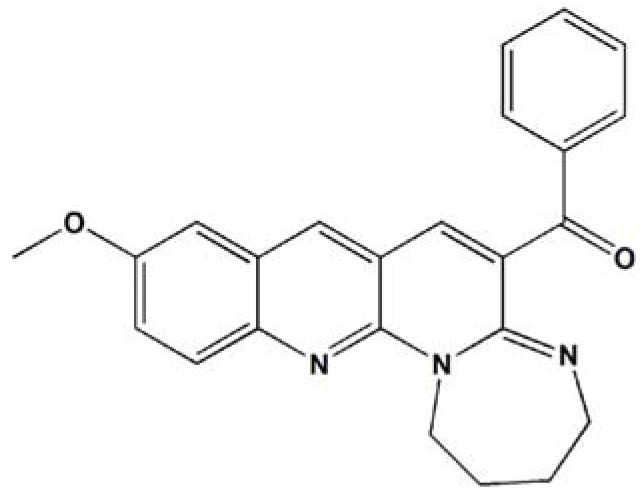
Chemical structure of naphthyridine derivative 3u.

Naphthyridine derivatives are a widely used class of heterocycles due to their pharmacological activities including blockade of enzymes [24], antagonism of receptors [25], and anticancer [26], antibacterial [27], anti-inflammatory [28], and antioxidant activity [29]. To screen more compounds with pharmacological activity, we built libraries of naphthyridine derivatives in a parallel way [30]. We evaluated several 1,3-diazaheterocycle fused [1-a][1,8]naphthyridine derivatives to ascertain the cytotoxicity in a series of cancer cell lines and found that the compound 3u (structure shown in Figure 1) showed good anticancer activity in human malignant melanoma cell line A375. In the current study, we showed the anticancer activity of 3u in nine kinds of cancer cell lines in comparison to the HSF normal cell line and explored the molecular mechanism of 3u-induced melanoma cell death preliminarily.

## 2. Results

### 2.1. 3u Decreases Cell Viability in Human Cancer Cells

**Figure 2:**
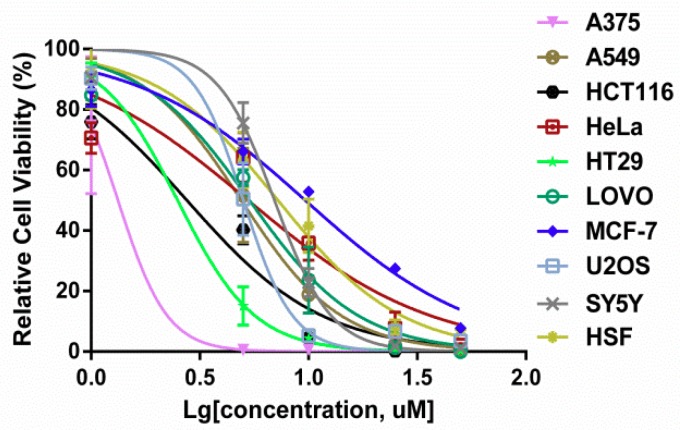
Cell viabilities of cell lines in response to 3u. The cell growth of nine cancer cell lines (A375, A549, HCT116, HeLa, HT29, LOVO, MCF7, SY5Y, and U2OS) and a normal cell line 9 (HSF) was measured using MTT assay 48 h after 3u exposure at various concentrations (0 µM, 1 µM, 5 µM, 10 µM, 25 µM, and 50 µM) (*n* = 3).

To assess the effect of 3u on the suppression of cell growth, nine cancer cells lines (A375, A549, HCT116, HeLa, HT29, LOVO, MCF7, SY5Y, and U2OS) and a normal cell line (HSF) were exposed to various concentrations of 3u (1 µM, 5 µM, 10 µM, 25 µM, and 50 µM) along with DMSO as a control for 48 h. The growth inhibitory activity was determined as cell viability curves (Figure 2) and IC_50_ values (Table 1) for each cell line via MTT assay. Compound 3u exhibited relatively good anticancer activity in A375 cells. At the same time, the cytotoxic effect of 3u in normal cell line HSF was lower than that of most of the cancer cell lines except MCF7. Therefore, we investigated the mechanism of the anticancer effect of 3u in A375 cells.

The results are shown as the mean ± SEM of three separate experiments.

### 2.2. 3u-Induced Apoptosis at High Concentrations of 3u Depending on Caspase-3 Was Not Caused by ER Stress or Mitochondrial Pathway

**Figure 3:**
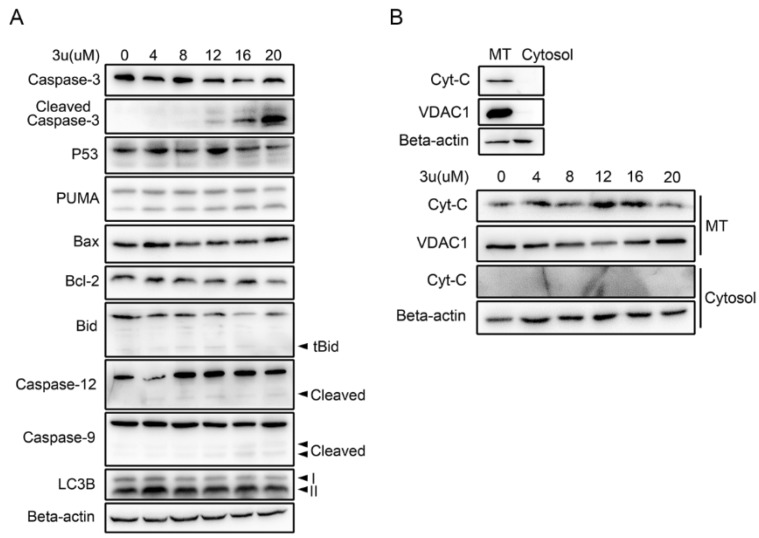
3u induced apoptosis at high concentrations of 3u depending on whether caspase-3 was caused by the ER-stress or mitochondrial pathways. After exposure to various concentrations of 3u for 6 h, (**A**) the proteins related to the ER-stress and mitochondrial pathways were examined along with caspase-3 using an immunoblot analysis in A375 cells. All of the protein expression levels were unchanged; (**B**) mitochondrial proteins and cytoplasmic proteins were separated completely (upper). 3u could not induce cytochrome c release in A375 cells (lower). MT: mitochondria.

Because caspase-dependent apoptosis is a common mechanism in chemotherapy, we first checked whether A375 cells triggered caspase-3 dependent apoptosis by 3u treatment. Therefore, cleaved caspase-3 was examined in 3u-treated A375 cells via Western blot analysis (Figure 3A). We found that caspase-3 cleaved at high concentrations of 3u (12 µM, 16 µM, and 20 µM) treated A375 cells. Three possible pathways can cause caspase-3 cleavage: the mitochondrial pathway via cytochrome c, the endoplasmic reticulum (ER)-stress pathway via caspase-12, and the DR-induced pathway via caspase-8. ER-stress can lead to the cleavage of caspase-12, which will then cleave caspase-3 [31]. When cells face DNA damage or reactive oxygen species (ROS), Mitogen-Activated Protein Kinase 8 (JNK) can potentially lead to an increase in the expression of pro-apoptotic genes (Bid, p53, and so on) and/or a decrease in the expression of prosurvival genes such as Bcl-2 [32]. The upregulation of p53 will lead to the subsequent upregulation of Bax and Bcl-2-Binding Component 3 (PUMA), inhibiting the antiapoptotic function of Bcl-2, and then MOMP will occur [33].

The expressions of p53, PUMA, Bax, and Bcl-2 were examined, but they were unchanged (Figure 3A). At the same time, autophagy did not appear because the ratio of Microtubule Associated Protein 1 Light Chain 3 Beta (LC3B)-I and II hardly altered (Figure 3A). Slight signals of truncated Bid (tBid), cleaved caspase-12 and cleaved caspase-9 were observed at all concentrations of 3u-treated A375 cells (Figure 3A). But it was not consistent with the cleavage of caspase-3, which was only observed at high concentrations. Possibly, these were just the background signal. We further explored whether cytochrome c (Cyt-C) was released from mitochondria. Cyt-C and Voltage Dependent Anion Channel 1 (VDAC1) existed only in mitochondrial proteins from untreated A375 cells (Figure 3B, upper). Hence, we successfully extracted mitochondrial protein from the whole lysis. However, the release of Cyt-C from mitochondria did not occur at all concentrations of 3u-treated A375 cells (Figure 3B, lower). Again, the results proved that slight signals of tBid and cleaved caspase-9 were just background signals.

### 2.3. 3u-Induced Apoptosis at High Concentrations of 3u Was Caused by the Upregulation of DRs and FADD

**Figure 4:**
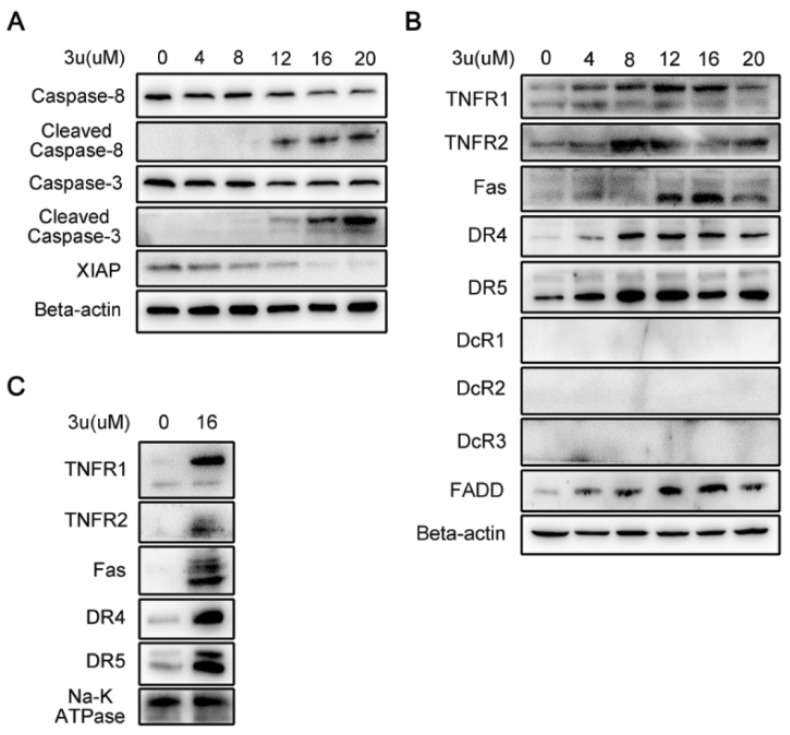
3u-induced apoptosis was caused by upregulation of DRs and FADD via the extrinsic pathway. After 6 h of treatment of 3u in A375 cells (**A**) the extrinsic-related proteins were determined by Western blot analysis; (**B**) DRs and scaffold protein FADD, which initiated the death signal, were examined via immunoblot; and (**C**) membrane proteins were extracted, and DRs were determined by a western blot analysis.

As the mitochondrial pathway and the ER-stress pathway were excluded from the mechanism of 3u-induced apoptosis, we certified whether apoptosis was triggered by the extrinsic pathway using immunoblot analysis. Compound 3u treatment induced the concentration-dependent cleavage of caspase-8 in A375 cells, which was consistent with the cleavage of caspase-3 at high concentrations of 3u (12 µM, 16 µM, and 20 µM) (Figure 4A). Thus, 3u-induced apoptosis was triggered via the extrinsic pathway. Moreover, X-Linked Inhibitor Of Apoptosis (XIAP), which could inhibit the cleavage of caspase-3 via its BIR2 domain [34,35], decreased in a dose-dependent manner and demonstrated an opposite variation tendency compared with cleaved caspase-3, which further promoted 3u-induced apoptosis (Figure 4A).

Except for specific ligand-binding-mediated activation, it is not surprising that high expression of DRs can lead to apoptosis by DRs self-activation. In addition, overexpression of the adaptor protein FADD can form large, filamentous aggregates, termed death effector filaments (DEFs) by self-association and initiate apoptosis independent of receptor cross-linking [36,37,38,39]. To further investigate how the cell death signal is initiated via the extrinsic pathway, we determined the protein expression level of DRs (TNFR1, TNFR2, Fas, DR4, DR5, DcR1, DcR2, and DcR3) and scaffold protein (FADD). All of the DRs and adaptor protein except the three decoy receptors (DcR1, DcR2, and DcR 3) demonstrated upregulation in a concentration-dependent manner (Figure 4B). Thus, the death signal initiated via the self-activation of DRs and upregulation of scaffold protein.

It was necessary to ascertain the subcellular localization of upregulated DRs in 3u-treated A375 cells for DRs were transmembrane proteins and functional only in membrane. Thus, membrane proteins were extracted from untreated and 16 µM 3u exposed A375 cells, and then Western blot analysis was performed. All five DRs were upregulated by 3u, and were located in the membrane (Figure 4C).

**Figure 5:**
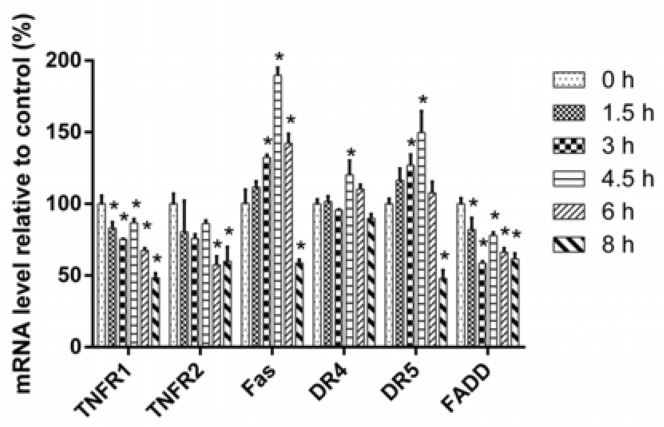
The relative mRNA expression results of DRs and FADD. Cells were treated with 16 µM of 3u for 0 h, 1.5 h, 3 h, 4.5 h, 6 h, and 8 h, and the mRNA relative expression level of DRs, FADD was detected by qPCR. The data were expressed as means ± SD (*n* = 3). *, *p* < 0.05 vs. 0 h group (unpaired Student’s *t*-test).

To illuminate the mechanism of upregulation of DRs and FADD, we examined the mRNA expression of these genes via qPCR. After treatment by 16 µM of 3u for 0 h, 1.5 h, 3 h, 4.5 h, 6 h, and 8 h, the total RNA of these A375 cells were extracted and reverse transcribed into cDNA. Then, a qPCR assay was performed to detect the mRNA relative expression level of DRs, FADD. Relative mRNA expression of Fas, DR4, and DR5 increased and reached a peak at 4.5 h but then decreased at 6 h and 8 h because of cell death. However, for TNFR1, TNFR2, and FADD, the overall trend was downward (Figure 5). Hence, the upregulation of Fas, DR4, and DR5 was mediated by transcription but for TNFR1, TNFR2, and FADD it was not via transcription but rather by post-transcription or translation.

### 2.4. The Mechanism of Cell Death Induced by Low Concentrations of 3u Was Not Apoptosis But Rather Necroptosis

**Figure 6:**
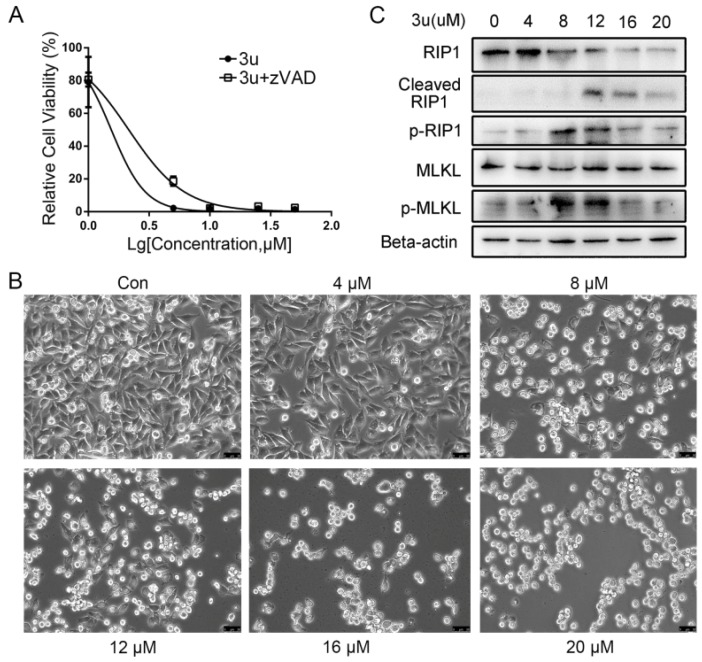
The mechanism of cell death induced by low concentrations of 3u was not apoptosis but rather necroptosis. In A375 cells (**A**) the cell viabilities in response to 3u did not differ greatly with or without pretreatment of 30 µM zVAD-fmk for 1 h. A375 cells were treated with various concentrations of 3u for 6 h, and then (**B**) cell morphological changes were observed under microscope (200×). Scale bar 50 µm; and (**C**) necroptosis-related proteins were examined via Western blot analysis.

To further certify that the 3u-induced cell death was apoptosis, A375 cells were treated with 30 µM caspase inhibitor zVAD-fmk and 3u at various concentrations (0 µM, 1 µM, 5 µM, 10 µM, 25 µM, and 50 µM). Surprisingly, compared with the cells treated with only 3u, IC_50_ of double-treated cells did not clearly increase (Figure 6A) (1.54 µM vs. 2.15 µM). Moreover, after exposure to 3u for 6 h, some A375 cells died even at low concentrations of 3u (4 µM and 8 µM) let alone at high concentrations (12 µM, 16 µM, and 20 µM) (Figure 6B). This is not consistent with the cleavage of caspase-8 and caspase-3, which cleaved only in cells treated with high concentrations of 3u (12 µM, 16 µM, and 20 µM) (Figure 4A). Thus, cell death at low concentrations of 3u was not induced by apoptosis.

The Activity of conventional death receptors of TNFR superfamily could initiate necroptosis [17,18]. As we observed previously, DRs and adaptor proteins were upregulated in the treatments with both high and low concentrations of 3u (Figure 4B). Therefore, we assumed that low concentrations of 3u triggered necroptosis, which led to cell death in A375 cells. To testify this assumption, RIP1 and MLKL which were related to necroptosis were examined via a Western blot analysis. RIP1 was cleaved by cleaved caspase-8 at high concentrations (Figure 6C), which was consistent with the cleavage of caspase-8. RIP1 and MLKL were phosphorylated not only at 8 µM but also at 12 µM, in which the former was stronger than the latter (Figure 6C). Phosphorylation of the two proteins at 4 µM, 16 µM, and 20 µM was almost the same as that at 0 µM. This result meant that necroptosis was inhibited at high concentrations of 3u via cleavage of RIP1. However, the inhibition at 12 µM was not enough to eliminate necroptosis completely. So apoptosis and necroptosis existed simultaneously at 12 µM. Eight micromolar of 3u induced necroptosis, which finally led to cell death. The upregulation of DRs and adaptor proteins at 4 µM was much weaker than at other concentrations, so necroptosis was close to background levels.

### 2.5. Caspase Inhibitor zVAD-fmk Promoted Necroptosis at High Concentrations of 3u While Inhibiting Apoptosis

**Figure 7:**
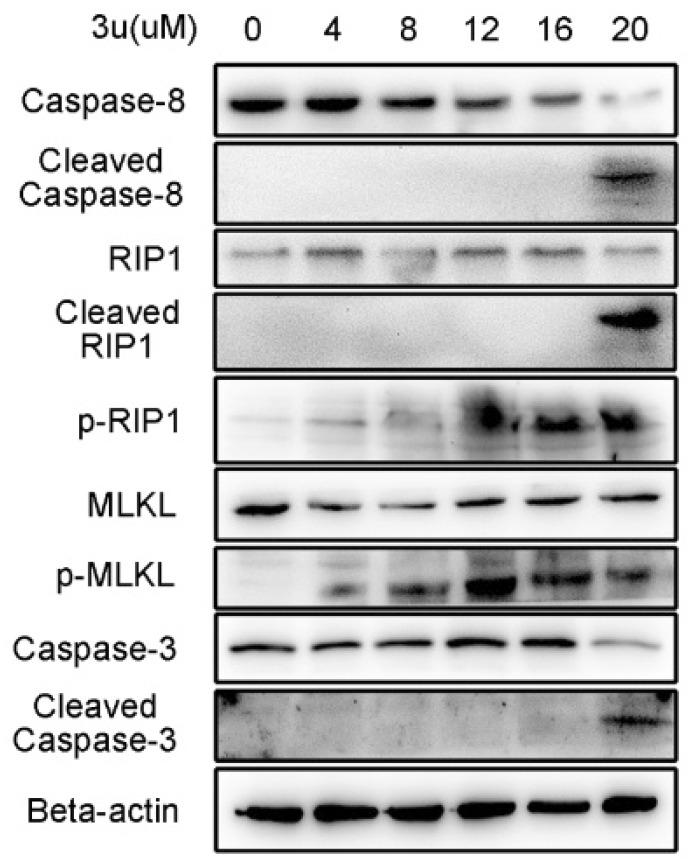
Caspase inhibitor zVAD-fmk promoted necroptosis at high concentrations of 3u while inhibiting apoptosis. Apoptosis- and necroptosis-related proteins were examined using Western blot analysis after a 30 µM zVAD-fmk pretreatment for 1 h and treatment with various concentrations of 3u for 6 h in A375 cells.

Although we observed that the IC_50_ hardly changed between 3u only and 3u+zVAD-fmk treated A375 cells via MTT assay, it was necessary to verify the biochemical changes of A375 cells under 3u+zVAD-fmk treatment via immunoblot. Caspase-8, RIP1 and caspase-3 were cleaved in only 20 µM of 3u and 30 µM zVAD-fmk treated A375 cells (Figure 7), which indicated that 3u induced apoptosis was completely inhibited by zVAD-fmk at 12 µM and 16 µM and only occurred at 20 µM.

Unlike A375 cells treated with only 3u, phosphorylation of RIP1 and MLKL was observed at 4 µM, 8 µM, 12 µM, 16 µM, and 20 µM in which the phosphorylation signal of both proteins at 12 µM was stronger than others and the signals at 16 µM and 20 µM were much stronger than the blank control (Figure 7). This result indicated that necroptosis was activated at 12 µM, 16 µM, and 20 µM because caspase-8 was inhibited by zVAD-fmk and could not disable the kinase function of RIP1 by cleaving it. Apoptosis and necroptosis occurred concurrently at 20 µM.

### 2.6. RIP1 Kinase Inhibitor Necrostatin-1 (Nec-1) Inhibited Necroptosis While Apoptosis Was Not Affected

**Figure 8:**
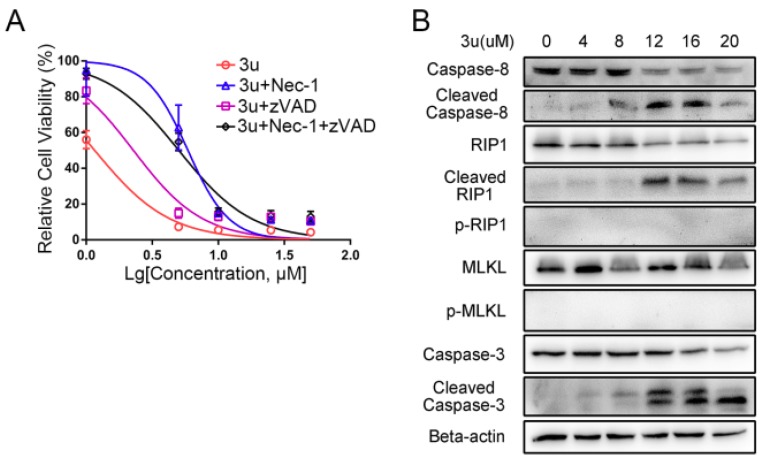
RIP1 kinase inhibitor Necrostatin-1 (Nec-1) inhibited necroptosis while apoptosis was not affected. (**A**) With or without pretreatment of 30 µM zVAD-fmk and/or 20 µM Nec-1 for 1 h, A375 cells were treated by different concentrations of 3u for 6 h, and cell growth was measured using MTT assay. (**B**) Apoptosis- and necroptosis-related proteins were examined using Western blot analysis after 20 µM Nec-1 pretreatment for 1 h and treatment with various concentrations of 3u for 6 h.

To further certify that 3u-induced cell death at low concentrations was necroptosis, A375 cells were treated by various concentrations of 3u (0 µM, 0 µM, 1 µM, 5 µM, 10 µM, 25 µM, and 50 µM), 30 µM zVAD-fmk and 20 µM RIP1 kinase inhibitor Nec-1, and an MTT assay was performed. More cells survived under the double treatment (3u+Nec-1) or triple treatment (3u+zVAD-fmk+Nec-1) than under the 3u treatment or 3u+zVAD-fmk double treatment (Figure 8A). The IC_50_ of the cells treated with 3u+Nec-1 was five-fold that of the 3u-treated cells (5.896 µM and 1.16 µM), while the IC_50_ of the cells treated with 3u+zVAD-fmk was double that of the 3u-treated cells (2.279 µM and 1.16 µM). The IC_50_ of the cells treated with 3u+zVAD-fmk+Nec-1 was slightly lower than in the cells treated with 3u+Nec-1 (5.029 µM and 5.896 µM). The results indicated that the inhibition of caspase-8 could not suppress 3u-induced cell death, while elimination of RIP1 kinase activity could survive 3u-treated cells to a certain extent. However, compared with the cells treated by 3u+Nec-1, the cells exposed to 3u+zVAD-fmk+Nec-1 could not survive at higher concentrations of 3u, which might be caused by the cytotoxic accumulation of the three compounds.

Then, the biochemical changes of A375 cells under 3u+Nec-1 treatment were determined via a Western blot analysis. The phosphorylation of RIP1 and MLKL could not be observed, while the cleavage of caspase-8, RIP1 and caspase-3 remained nearly unchanged compared with the 3u-treated cells (Figure 8B and Figure 4A). Slight signals of cleaved caspase-8 and caspase-3 at 4 µM and 8 µM were observed, which might be due to the cytotoxicity of Nec-1. Thus, Nec-1 inhibited RIP1 kinase activity. 3u-induced necroptosis at low concentrations was suppressed, but apoptosis at high concentrations was not influenced.

## 3. Discussion

A few years ago, it was believed that efficient anticancer regimens would either eliminate cancer cells by inducing apoptosis or constantly arrest them in the G1 phase of the cell cycle [6]. Unfortunately, genetic mutations and/or abnormal expression of apoptosis-related genes, such as FLIP, p53, and caspase [40], cause anticancer drug resistance. Recently, some anticancer reagents have been found to induce cancer cell death not via apoptosis but rather via other forms of cell death, such as necroptosis or mitotic catastrophe-engaged apoptosis [41], which could overcome the conventional drug resistance.

In the current study, we assessed the anticancer activity of a 1,3-diazaheterocycle-fused [1,2-a][1,8] naphthyridine derivative 3u and found that it demonstrated good anticancer activity: the IC_50_ of the normal cell line HSF was lower than for all cancer cell lines except MCF7. It was reported that MCF7 lost caspase-3 because of a 43-base pair deletion within exon 3 of the *CASP-3* gene [42], which was the same as we observed previously [43]. MCF7 cells are still sensitive to TNF [42] but resistant to TRAIL induced cell death [43]. 3u can upregulate TNFR1, TNFR2, DR4, DR5, and FADD, so MCF7 is not as sensitive as other cancer cell lines to 3u. It was reported that Fas, DR4, and DR5 were directly regulated by p53 when treated with DNA damage reagents [44,45,46,47]. But here, upregulation of p53 and its target proteins (PUMA and Bax) was not observed, so high expression of Fas, DR4, and DR5 was not mediated by p53. The mechanism of upregulation of these five DRs and FADD remained unknown and require further research.

**Figure 9:**
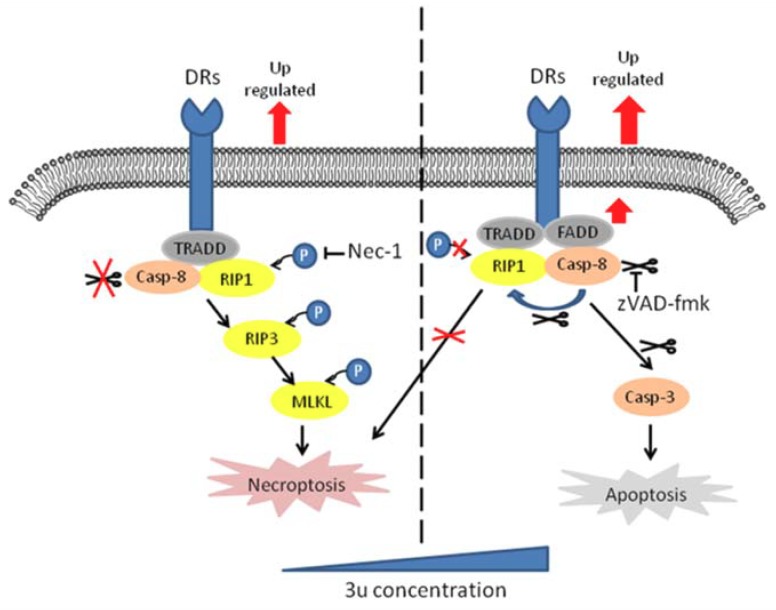
Mechanism of 3u-induced cell death in A375 cells. The model shows that 3u induces necroptosis at low concentrations and apoptosis at high concentrations in human melanoma A375 cells, and the activity of caspase-8 plays a key role in deciding the PCD type at high concentrations of 3u.

Caspase-8-mediated cleavage of RIP1 triggers the caspase cascade and induces apoptosis [19]. When caspase-8 is absent or inhibited, kinase-active RIP1 recruits and activates RIP3 and then phosphorylates MLKL, which induces necroptosis [21,22,48]. We found that 3u could induce caspase-3-dependent apoptosis in A375 cells at high concentrations (16 µM and 20 µM) via the activation of caspase-8, which was initiated by the upregulation of DRs and scaffold protein. At low concentrations of 3u (8 µM), necroptosis occurred because the upregulation of DRs and scaffold protein was weaker at low concentrations than at high concentrations. Twelve micromolar was a critical concentration because both necroptosis and apoptosis appeared at this concentration (Figure 4B). Thus sufficient activity of caspase-8 could accumulate to trigger apoptosis. ZVAD-fmk could suppress apoptosis and extend necroptosis at high concentrations of 3u. Nec-1 could inhibit necroptosis but could not influence apoptosis. Based on the above results, the activity of caspase-8 played a key role in determining the PCD type at high concentrations of 3u in A375 cells (Figure 9).

Currently, targeted therapy has become a new therapeutic strategy in melanoma treatment. BRAF, a member of the RAF family, often mutates at the V600 codon in melanoma (V600E, 80% of mutations; V600K, 16% of mutations; and V600D/R, 3% of all mutations in melanoma) [49,50]. In most cases, BRAF-resistant melanomas bear additional mutations, reactivating the MAPK pathway [51]. However, the first BRAF inhibitor tested in patients with melanoma, sorafenib, showed little efficacy either alone [52] or combined with other conventional chemotherapeutic agents [53]. The association of BRAF and MEK inhibitors (dabrafenib and trametinib) was less toxic and more effective than BRAF monotherapy [54,55]. A combination of targeted therapy and conventional chemotherapy is still a promising anticancer strategy.

## 4. Materials and Methods

### 4.1. Chemicals and Antibodies

3u compound (10-Methoxy-1,2,3,4-tetrahydrobenzo(*g*)(1,3)diazepino (1,2-a)-(1,8)naphthyridin-6-yl)(phenyl) methanone was synthesized as previously described [30] at the Key Laboratory of Medicinal Chemistry for Natural Resources, Ministry of Education, School of Chemical Science and Technology, Yunnan University, Kunming, China. Compound 3u was dissolved in DMSO and prepared as 33 mM stock solution, and its aliquots were stored at −20 °C. Caspase inhibitor zVAD-fmk and RIP1 kinase inhibitor Necrostatin-1 (Nec-1) were from MedChemExpress (Monmouth Junction, NJ, USA). Tween-20, sodium dodecyl sulfate, and TEMED were from Amresco (Solon, HO, USA). Glycine, dithiothreitol, and Tris-base were from Solarbio (Beijing, China). General biochemicals and chemicals were purchased from Thermo Fishier Scientific (Waltham, MA, USA), Merck (Whitehouse Station, NJ, USA), or Sigma Chemical Co. (St. Louis, MO, USA).

The antibodies for immunoblotting were from the following sources. Apoptosis/Necroptosis Antibody Sampler Kit (#92570), Death Receptor Antibody Sampler Kit (#8356) (except DcR2, DR5 and FADD), rabbit anti-DcR1 (#4756), rabbit anti-DcR2 (#4741), rabbit anti-Bcl-2 (#2870), rabbit anti-XIAP (#2045), mouse anti-caspase-9 (#9508), and rabbit anti-caspase-12 (#2202) were from Cell Signaling Technology (Beverly, MA, USA); mouse anti-FADD (#F8053) was from Sigma Chemical Co.; rabbit anti-DR4 (ab8414), rabbit anti-DR5 (ab47179), rabbit anti-Bax (ab32503), rabbit anti-cytochrome c (ab133504), rabbit anti-LC3B (ab51520), rabbit anti-sodium potassium ATPase (ab76720), rabbit anti-VDAC1/Porin (ab154856), and rabbit anti-PUMA (ab33906) were purchased from Abcam (Cambridge, MA, USA); and mouse anti-p53 (sc-126), mouse anti-beta actin (sc-47778), and mouse anti-Bid (sc-56025) were obtained from Santa Cruz Biotechnology (Dallas, TX, USA).

### 4.2. Cell Lines and Culture Conditions

The cancer cell lines A549, HCT116, HeLa, HT29, LOVO, MCF7, SY5Y, and U2OS were purchased from the American Type Culture Collection (Manassas, VA, USA). A375 and the normal cell line HSF were purchased from the Cell Bank Culture Collection of the Kunming Institute of Zoology, Chinese Academy of Sciences (Kunming, China). A549, HCT116, HT29, LOVO, and MCF7 were maintained in RPMI-1640 medium from Hyclone (Logan, UT, USA). HeLa, SY5Y, U2OS, and HSF were cultured in Dulbecco’s modified minimal essential medium (DMEM) (Hyclone). All culture media were supplemented with 10% fetal bovine serum (Hyclone). Cells were grown in a humidified incubator at 37 °C under an atmosphere of 5% CO_2_ and subcultured with 0.25% trypsin-EDTA when ~80% confluence was reached.

### 4.3. Cell Viability Assay

Cells were seeded in 96-well cell culture plates at 1 × 10^4^ per well, allowed to adhere overnight, and treated the next day with 3u at various concentrations (1 µM, 5 µM, 10 µM, 25 µM, and 50 µM) along with DMSO as a control for 48 h. Then, the cells were added with 100 µg per well of 3-(4,5-dimethylthiazol-2-yl)-2,5-diphenyltetrazolium bromide (MTT) and incubated for another 4 h at 37 °C. The medium was aspirated and changed to 150 µL DMSO. After 15 min of incubation, the absorbance was measured at 490 nm using a VICTOR X5 Multilabel Plate Reader from Perkin-Elmer (Waltham, MA, USA). The concentrations of the compounds causing 50% reduction of cell viability (IC_50_) were calculated. The experiments were performed at least in triplicate.

### 4.4. Extraction of Mitochondrial and Membranous Proteins

Eight-million A375 cells were cultured in 10 cm culture dishes and treated with 3u compound for 6 h were harvested and washed once with ice-cold PBS. Mitochondrial and cytoplasmic proteins were extracted using a Cell Mitochondria Isolation Kit (Beyotime Biotechnology, Shanghai, China), and membranous proteins were isolated using a Membrane and Cytosol Protein Extraction Kit (Beyotime Biotechnology) following the manufacturer’s instructions.

### 4.5. SDS Electrophoresis and Immunoblotting

For immunoblotting, A375 cells were seeded at a density of 8 × 10^5^ cells in 6 cm culture dishes, allowed to adhere overnight and treated with 3u compound for 6 h. Then, the cells were washed once with PBS, pelleted and lysed in the lysis buffer (50 mM Tris (pH 7.4), 150 mM NaCl, 1% Triton X-100, 1% sodium deoxycholate, 0.1% SDS), and an EDTA-free protease inhibitor cocktail tablet (Roche, Mannheim, Germany) was added. The protein concentration was determined using an Enhanced BCA Protein Assay Kit (Beyotime Biotechnology). Equal quantities of cell lysate protein (20 ug) were separated by 12% SDS polyacrylamide gel electrophoresis and blotted onto PVDF membranes (Millipore Corp., Billerica, MA, USA). Membranes were blocked with 10% nonfat milk (Becton Dickson, NJ, USA) in Tris-buffered saline-Tween-20 buffer (10 mM Tris, 100 mM NaCl, 0.1% Tween-20) for 1 h at room temperature. The blots were probed overnight at 4 °C with a primary antibody followed by horseradish-peroxidase-conjugated anti-mouse or anti-rabbit IgG antibodies (Thermo Fishier Scientific) for 1.5 h at room temperature. The epitope was detected with Immobilon Western Chemiluminescent HRP Substrate from Millipore (Whitehouse Station, NJ, USA). Chemiluminescent signals were detected and analyzed using a Tanon-5500 chemiluminescent imaging system (Tanon, Shanghai, China).

### 4.6. Quantitative Real-Time Polymerase Chain Reaction (qPCR)

For qPCR, A375 cells were seeded at a density of 8 × 10^5^ cells in 6 cm culture dishes, allowed to adhere overnight, and treated with 16 µM 3u compound for 0 h, 1.5 h, 3 h, 4.5 h, 6 h, and 8 h. Total RNA was extracted using Trizol reagent (Takara, Shiga, Kusatsu, Japan) and then reverse transcribed for cDNA synthesis with an HiScript Q RT Supermix for qPCR (Vazyme, Nanjing, China) following the manufacturer’s protocol. QPCR was performed in 12 µL reactions using FastStart SYBR Green Master Mix (Roche, Basel, Switzerland). The target mRNA expression level was normalized to the beta-actin (actb). The forward and reverse primers used in qPCR were actb (5′-CATGTACGTTGCTATCCAGGC-3′ and 5′-CTCCTTAATGTCACGCACGAT-3′); Fas (5′-AGTGGAAATAAACTGCACCCGGA-3′ and 5′-CCTCTTTGCACTTGGTGTTGCTG-3′); TNFR1 (5′-CCTAGGGGACAGGGAGAAGAGAG-3′ and 5′-TCTGAGGTGGTTTTCTGAAGCGG-3′); TNFR2 (5′-CACCGGGAGCTCAGATTCTTCC-3′ and 5′-TGTGTCTCCCATTGTGGAGCTG-3′); DR4 (5′-TGGCACACAGCAATGGGAACATA-3′ and 5′-TGGTGCAGGGACTTCTCTCTTCT-3′); DR5 (5′-CAAGACCCTTGTGCTCGTTGTC-3′ and 5′-GCAGGAGATGCAATCTCTACCGT-3′); FADD (5′-ACACCAAGATCGACAGCATCGAG-3′ and 5′- GAACCTCTTGTACCAGGTCAGCC-3′).

### 4.7. Statistical Analysis

The data were expressed as the means ± SEM from experiments that were performed on at least three separate occasions for the cell viability MTT assay. The IC_50_ was calculated using GraphPad Prism 6.01 from Graphpad Software (La Jolla, CA, USA). Comparisons between groups in qPCR for difference were performed with unpaired Student’s *t*-test. A *p* value ≤ 0.05 was considered significant.

## Figures and Tables

**Table 1:** IC_50_ values of 3u on the growth inhibition of cell lines.

Compound	Cell Lines IC_50_ (µM)				
3u	A375	A549	HCT116	HeLa	HT29
	1.52 ± 0.10	3.69 ± 0.63	2.54 ± 0.20	4.31 ± 0.71	2.45 ± 0.13
	LOVO	MCF7	U2OS	SY5Y	HSF
	5.72 ± 0.37	10.24 ± 1.03	4.70 ± 0.76	4.60 ± 0.64	7.27 ± 0.03
